# PRDM16, a new kid on the block in cardiovascular health and disease

**DOI:** 10.1093/cvr/cvaf089

**Published:** 2025-05-29

**Authors:** Jore Van Wauwe, Hannelore Kemps, Pieter Vrancaert, Alexia Mahy, Robin Schellingen, Mandy O J Grootaert, Manu Beerens, Aernout Luttun

**Affiliations:** Center for Molecular and Vascular Biology, Department of Cardiovascular Sciences, KU Leuven, Campus Gasthuisberg, Onderwijs & Navorsing 1, Herestraat 49, box 911, 3000 Leuven, Belgium; Cambridge Stem Cell Institute, University of Cambridge, Cambridge, UK; Center for Molecular and Vascular Biology, Department of Cardiovascular Sciences, KU Leuven, Campus Gasthuisberg, Onderwijs & Navorsing 1, Herestraat 49, box 911, 3000 Leuven, Belgium; Center for Molecular and Vascular Biology, Department of Cardiovascular Sciences, KU Leuven, Campus Gasthuisberg, Onderwijs & Navorsing 1, Herestraat 49, box 911, 3000 Leuven, Belgium; Center for Molecular and Vascular Biology, Department of Cardiovascular Sciences, KU Leuven, Campus Gasthuisberg, Onderwijs & Navorsing 1, Herestraat 49, box 911, 3000 Leuven, Belgium; Center for Molecular and Vascular Biology, Department of Cardiovascular Sciences, KU Leuven, Campus Gasthuisberg, Onderwijs & Navorsing 1, Herestraat 49, box 911, 3000 Leuven, Belgium; Center for Molecular and Vascular Biology, Department of Cardiovascular Sciences, KU Leuven, Campus Gasthuisberg, Onderwijs & Navorsing 1, Herestraat 49, box 911, 3000 Leuven, Belgium; Endocrinology, Diabetes and Nutrition, UCLouvain, Avenue Hippocrate, box 55, 1200 Brussels, Belgium; Institute for Clinical Chemistry and Laboratory Medicine, University Medical Center Hamburg-Eppendorf, Hamburg, Germany; German Centre of Cardiovascular Research (DZHK), Partner Site Hamburg, Lübeck, Kiel, Hamburg, Germany; Center for Molecular and Vascular Biology, Department of Cardiovascular Sciences, KU Leuven, Campus Gasthuisberg, Onderwijs & Navorsing 1, Herestraat 49, box 911, 3000 Leuven, Belgium

**Keywords:** PRDM16, Transcription factors, Cardiomyopathy, Vascular disease, Cell-fate decision

## Abstract

Transcriptional regulation is essential for the development, homeostasis, and function of all organisms. Transcription factors and epigenetic modifiers play an indispensable role by direct or indirect interaction with DNA or chromatin. Although the role of transcription factor PRDM16 in adipose, haematopoietic, skeletal, and neural cell lineage specification is well-documented, its function within the cardiovascular system has only recently gained significant attention. Similar as in adipose tissue, PRDM16 displays an asymmetric expression pattern within the cardiovascular system, where it is exclusively expressed by ventricular cardiomyocytes and endothelial and smooth muscle cells of arteries while being absent in their atrial and venous counterparts. Concordantly, an increasing number of clinical and preclinical studies have identified PRDM16 as an important multi-modal regulator of cardiovascular development and function. Moreover, aberrant PRDM16 expression has now been linked to (cardio)vascular diseases, including left ventricular non-compaction, migraine, and coronary artery disease. In this review, we give a synopsis of PRDM16's expression and function within (developing) cardiovascular tissues and provide insights into how impaired PRDM16 signalling contributes to cardiovascular disease.

## Graphical Abstract

**Graphical Abstract:**
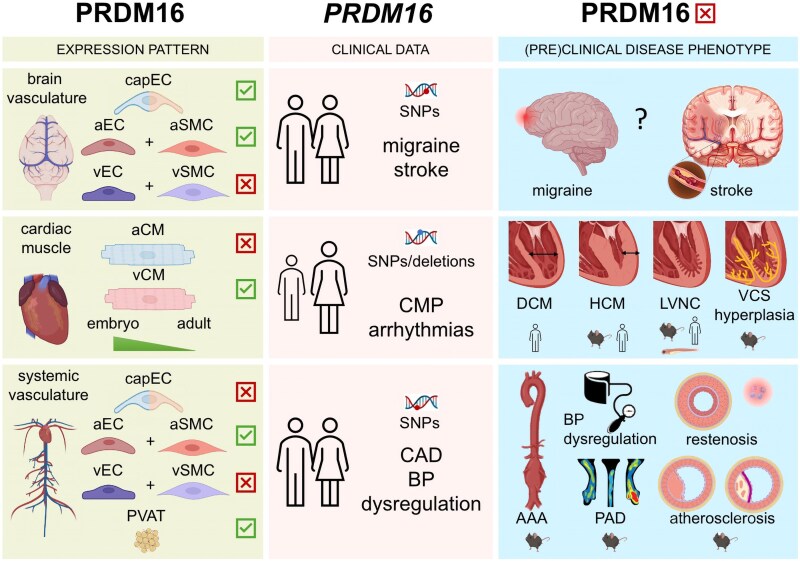

## 1. Introduction

The cardiovascular system is the first organ to develop and remains critical throughout our lifespan. Diseases affecting its formation and function remain a leading cause of death worldwide. The identification of the molecular mechanisms underlying cardiovascular diseases (CVDs) and their therapeutic and diagnostic exploration therefore represent an urgent clinical need. Many of these disease-causing mechanisms are associated with aberrant transcriptional regulation, a critical biological process that carefully titrates gene expression in a spatiotemporal fashion to orchestrate proper tissue, organ, and organism development and function. Key regulators indispensable in this process are transcription factors (TFs) and epigenetic modifiers, which interact directly or indirectly with DNA regulatory sequences and chromatin to modify gene expression.

**Figure 1:**
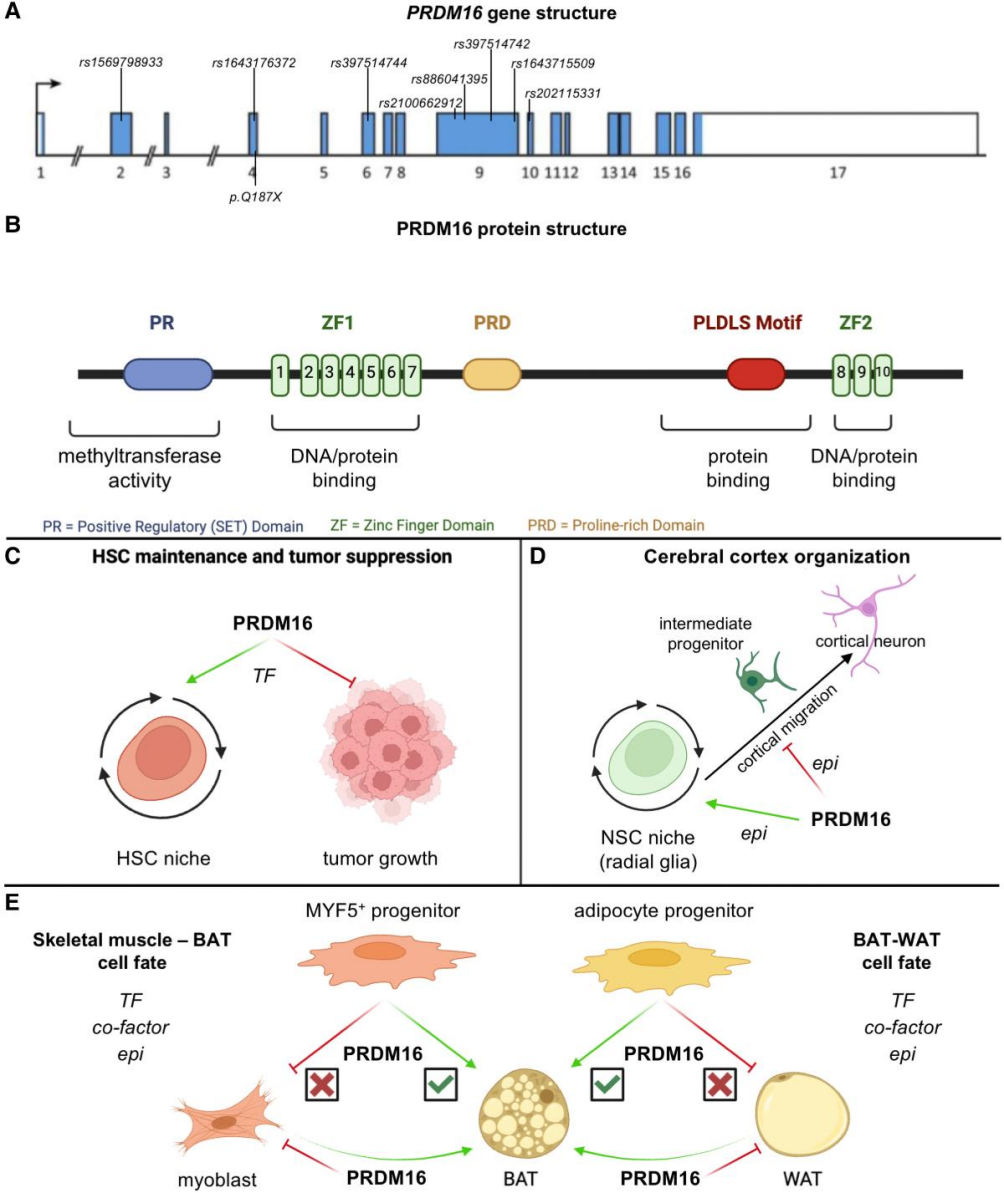
PRDM16 protein structure and mode of action outside the cardiovascular system. (*A*, *B*) The structure of the *PRDM16* gene, consisting of 17 exons (*A*) and the corresponding protein structure (*B*). Pathogenic variants listed in *Table 1* associated with cardiomyopathy are indicated. (*C*) PRDM16 is known for its transcriptional activator (green arrow) role in haematopoietic stem cell (HSC) maintenance and its repressor (red T-bar) role in cancer. (*D*) In the nervous system, PRDM16 functions as an epigenetic (epi) regulator to organise cells of the cerebral cortex. Hereto, it changes the epigenetic status of neural stem cells (NSCs, i.e. radial glia cells), thereby affecting intermediate progenitor formation and cortical neuron migration. (*E*) PRDM16 is best known for its role as binary cell-fate decision-maker in adipose tissue, where it drives the differentiation of brown adipose tissue (BAT), thereby repressing the transcriptional programme of white adipose tissue (WAT). Expression of PRDM16 in adipose progenitors or MYF5+ myocyte progenitors results in a BAT cell fate. Moreover, overexpression in myoblasts or WAT results in an identity switch of these cell types towards a more BAT-like phenotype. In this myogenic-adipocyte cell fate, PRDM16 functions as a transcription factor (TF), co-factor or epigenetic regulator. The figure was designed in BioRender and panel *A* was adapted from Ref.^8^

Here, we shed light on positive regulatory domain containing (PRDM)16, for which a prominent role in the cardiovascular system has recently emerged. PRDM16 belongs to a family of 17 TFs, known for their roles in driving and maintaining cell state transitions and, accordingly, their aberrant expression often leads to developmental defects or cancer.^1^ PRDM16 is one of the best-characterised PRDM family members and was first described as an oncogene in the context of leukaemia.^2^ There are three and four PRDM16 isoforms in mice and humans, respectively, generated by the presence of an alternative promoter or through alternative splicing.^3^ Expression driven by the alternative promoter results in a short PRDM16 protein (sPRDM16) which lacks the PR domain. sPRDM16 displays increased expression in leukaemic cells and has been extensively associated with leukaemogenesis, indicating that the PR domain of PRDM16 functions as an important repressor of cell division, tumour initiation, and growth.^2,4–7^ The full PRDM16 protein is encoded by 17 exons and includes, in addition to the N-terminal PR domain, the following functional domains: (i) a proline-rich domain, (ii) an SAM-binding motif PLDLS domain, and (iii) 2 zinc finger (ZF) regions, i.e. ZF1 and ZF2 containing 7 and 3 ZFs, respectively (*Figure 1A* and *B*). Via these domains, PRDM16 is involved in numerous cellular processes including lipid metabolism, regulation of mitochondrial dynamics, glucose homeostasis, and control of oxidative stress.^9^ Moreover, PRDM16 has been introduced as a novel small mothers against decapentaplegic (SMAD)-binding protein and a modulator of the transforming growth factor (TGF)-β pathway during embryonic development.^8,10^

Functionally, PRDM16 regulates cell differentiation in different tissues and serves as a main driver of brown/beige adipose lineage specification^8,11^ and craniofacial development.^12^ PRDM16 is also responsible for the maintenance and survival of foetal liver HSCs,^13,14^ and is required to sustain the capacity of HSCs to self-renew in both the foetal liver and in adult bone marrow, the latter at least in part by promoting their quiescence.^15^ Likewise, PRDM16 is required for the maintenance of foetal and adult neural stem cells (NSCs) and their differentiation towards ependymal cells.^14,16^ In NSCs, loss of PRDM16 increases reactive oxygen species (ROS) levels causing oxidative stress and compromising their function and survival.^14^ Moreover, PRDM16 in NSCs was recently shown to co-repress their proliferation in conjunction with bone morphogenetic protein (BMP) signalling.^17^ To achieve its role in HSC/NSC behaviour, several modes of action have been suggested (*Figure 1C* and *D*). First, based on the identification of putative DNA-binding sequences for PRDM16, it was demonstrated that sPRDM16 affects HSC self-renewal by direct binding as a TF to DNA (*Figure 1C*).^2^ Later during cortical neurogenesis, PRDM16 orchestrates cerebral cortex organisation, by acting as an epigenetic (co-)factor that docks on distal enhancers to change the epigenetic state of a population of cortical NSCs (called radial glia) to activate genes involved in intermediate progenitor cell production and repress genes related to cell migration, the latter through the intrinsic histone methyltransferase (HMT) activity of its PR domain (*Figure 1D*).^18^

The role of PRDM16 has been most extensively studied in adipose tissue where it is exclusively expressed in brown (BAT) or beige but not white adipose tissue (WAT). Consistent with this expression pattern, PRDM16 regulates the differentiation of adipocyte progenitors into BAT by repressing the WAT gene signature in adipocyte precursors^19,20^ and the gene programme of skeletal myoblasts in myogenic factor-5 (MYF5)-expressing precursors to favour BAT lineage identity.^11^ Within fat tissue, PRDM16 operates according to a multi-modal action plan (*Figure 1E*). While direct binding of its ZF domains to DNA drives expression of some BAT-specific genes and represses genes related to the response to interferon (IFN)γ, PRDM16 rather acts as an indirect transcriptional co-factor for the majority of genes enriched in either BAT or WAT. Indeed, while binding of PRDM16 to C-terminal-binding proteins (CtBPs) is essential for the suppression of the WAT-specific transcriptional programme, its complexation with CCAAT/enhancer-binding (C/EBP)β and peroxisome proliferator-activated receptor γ-co-activator (PGC)1α is required to activate the BAT-specific transcriptional programmes.^11,19–22^ During maintenance of BAT fate in adult mice and myoblast-to-BAT conversion, PRDM16 operates as an epigenetic co-factor, by recruiting euchromatic histone-lysine N-methyltransferase (EHMT)1.^23^ PRDM16 was later also shown to associate with MED1, a component of the Mediator complex which mostly binds to enhancers of BAT-selective genes which subsequently leads to three-dimensional chromatin architectural changes and their enhanced transcription.^24,25^ While PRDM16 also has its own intrinsic HMT capacity through its PR domain,^26^ it remains to be determined to what extent this contributes to its role during BAT development and maintenance,^8^ as the repression of genes related to the response of BAT to IFNγ by PRDM16 was independent of its methylation activity.^22^

Interestingly, PRDM16 displays a comparable asymmetric expression pattern within the cardiovascular system. We and others have indeed shown that PRDM16 is exclusively expressed in arterial endothelial cells (ECs) and smooth muscle cells (SMCs) of the vascular tree, while being absent in cells that form the venous vascular wall.^27–30^ Additionally, PRDM16 expression is present in ventricular but not atrial cardiomyocytes (CMs) of the mammalian heart.^12,29,31–33^ As a result, the role of PRDM16 in cardiovascular development, specification, and function has gained significant interest, with recent preclinical and clinical studies indicating an indispensable role for PRDM16 within the cardiovascular system. In this review, we zoom in on the expression pattern and function of PRDM16 in the cardiovascular system and discuss its role in CVDs.

## 2. Expression of PRDM16 and its role in cell-fate decision in the cardiovascular system

### 2.1 PRDM16 expression in the heart

**Figure 2:**
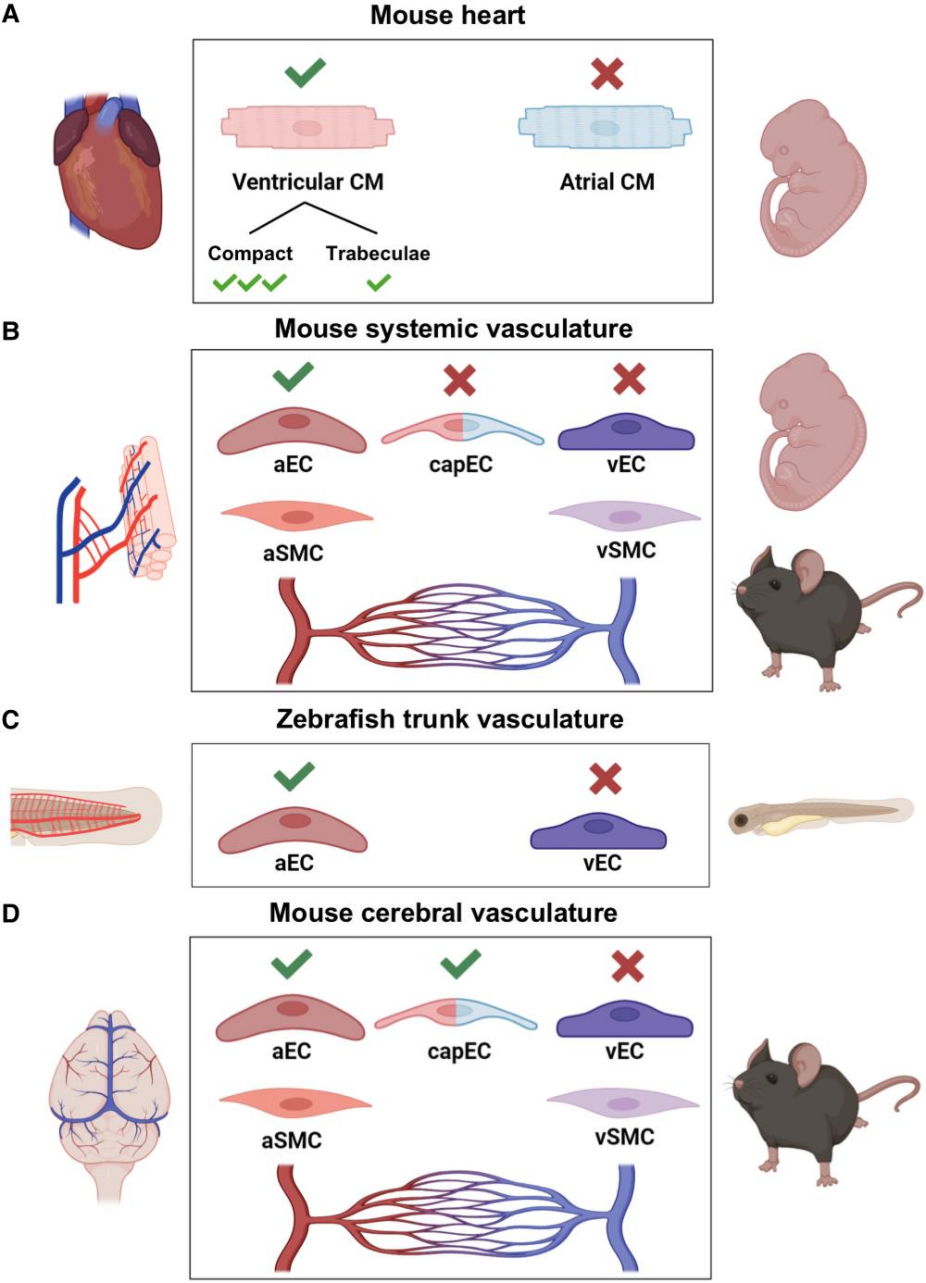
PRDM16 has an asymmetric expression pattern in the cardiovascular system. (*A*) In the developing mouse heart, PRDM16 expression is only detected in ventricular cardiomyocytes (CMs) while it is absent from atrial CMs. Moreover, within the ventricle, PRDM16 is more expressed in the compact myocardium compared to the trabecular zone. (*B*) In the systemic vasculature, we and others have shown that PRDM16 expression is restricted to arterial endothelial cells (aECs) and smooth muscle cells (aSMCs), whilst being absent from capillary (cap)ECs, venous (v)ECs, and vSMCs. (*C*) In accordance with its expression within the mouse vascular system, prdm16 is only expressed in aECs, but not in vECs of the tail axial vessels of zebrafish embryos. (*D*) In the cerebral vasculature of adult mice, PRDM16 expression is detected in aECs and aSMCs, and capECs, but is absent in vECs and vSMCs. The figure was designed in BioRender.

Multiple independent studies documented the conserved expression of PRDM16 in zebrafish, *Xenopus*, mouse, and human hearts.^29,31,33,34^ Using *Prdm16^LacZ/WT^* reporter mice, Bjork and colleagues were the first to report the asymmetric expression of *Prdm16* in ventricular vs. atrial CMs (*Figure 2A*).^12^ Additionally, PRDM16 was also found to be expressed in arterial ECs and SMCs of the (developing) coronary arteries.^35–38^ Horn *et al.* showed that *Prdm16* expression in the developing mouse heart first appears at embryonic day (E)10.5 in the left ventricle and later expands to both ventricles.^29^ These findings have now been confirmed in embryonic and adult hearts of mice and humans,^31,35,39–42^ despite the fact that cardiac PRDM16 levels decline postnatally.^33,39^ Spatial transcriptomics revealed that *Prdm16* expression in CMs is largely confined to the outer ventricular myocardium (i.e. the compact layer) in the developing mouse embryo, with a rather limited expression in the inner trabecular zone,^39^ which we recently confirmed (*Figure 2A*).^33^

### 2.2 PRDM16 expression and its role in the developing and early postnatal vasculature

Using comparative transcriptional profiling of human and mouse arterial and venous ECs, we reported PRDM16 as an arterial EC-enriched TF in the vasculature and later showed that this binary expression pattern extends to the SMC layer.^27,43^ In mouse embryos, PRDM16 expression is detected in the aorta around E9.5^43^ and continues to be exclusively expressed in arteries throughout development (*Figure 2B*). This arterial-restricted expression pattern was also maintained in adult mice, with strong expression in ECs and SMCs from both elastic conduit and smaller calibre muscular arteries.^28^ PRDM16 expression in retinal vessels was observed in both EC and SMC nuclei and was abruptly abolished in ECs from where arterioles connect to capillaries (*Figure 2B*).^30^ We and others further confirmed that prdm16 is present in zebrafish ECs at the earliest time points of vascular development and that its expression is—similar to mice and humans—confined to the arterial endothelium (*Figure 2C*).^43,44^ Hence, PRDM16 universally marks ECs and SMCs of arteri(ol)es independent of species, age, anatomic location, vessel calibre, or artery type, except in the brain vasculature, where its expression is not limited to (large) arteries and arterioles. In fact, *Prdm16* is enriched in brain capillary ECs when compared to those from different organs, and can even be detected at the venous side of the capillary bed (*Figure 2D*).^45–48^ The underlying triggers for this heterogeneous PRDM16 expression pattern and its functional consequences remain to be uncovered.

Knockdown experiments in zebrafish embryos demonstrated that prdm16 is required for developmental angiogenesis as shown by impaired intersegmental vessel formation in *prdm16* morphants.^43,49^ Accordingly, *PRDM16* knockdown in human induced pluripotent stem cell (iPSC)-derived ECs resulted in impaired migration,^49^ and EC-specific *Prdm16* deletion in mice from birth onwards compromised retinal angiogenesis by impairing EC migration.^44^ Thompson *et al*. further reported that EC-specific *Prdm16* deletion in mice from embryonic development onwards results in elevated *angiopoietin2* (*Angpt2)* expression leading to reduced SMC recruitment around the intima of the dorsal aorta (DA), suggesting a role for PRDM16 in developmental arteriogenesis.^44^ Using cultured human ECs, we demonstrated that PRDM16 drives arterial EC lineage specification, while results in zebrafish and mice by us and others indicated that PRDM16 is essential to suppress the alternate venous lineage programme *in vivo*.^43,44^ Additionally, we discovered that prdm16 synergises with canonical notch signalling and that loss of prdm16 alone, or in combination with impaired notch signalling, resulted in arteriovenous malformations in zebrafish.^43^ These findings are in line with our observations in human umbilical vein ECs (HUVECs), where forced expression of PRDM16 results in elevated mRNA levels of many arterial-specific genes, including genes involved in the NOTCH pathway.^43^ PRDM16 likely also arterialises ECs via NOTCH-independent mechanisms, as the arterialising power of PRDM16 exceeded that of 7 other arterial-specific TFs, including HEY2, the downstream TF in the NOTCH signalling cascade.^27^ Accordingly, enhanced expression of some arterially enriched genes in PRDM16-overexpressing HUVECs persisted in the presence of a canonical NOTCH inhibitor.^43^

Altogether, these studies indicate the indispensable role of PRDM16 in EC differentiation and (arterial) vascular development, lineage specification, and function, at least in part through its interaction with NOTCH signalling. The latter seems to be a consistent feature of PRDM16, as a context-dependent interaction between this TF and the NOTCH signalling cascade has also been reported in the neuronal system of both mice and fruit flies.^50,51^

## 3. Preclinical evidence for the role of PRDM16 in cardiovascular function and disease

**Figure 3:**
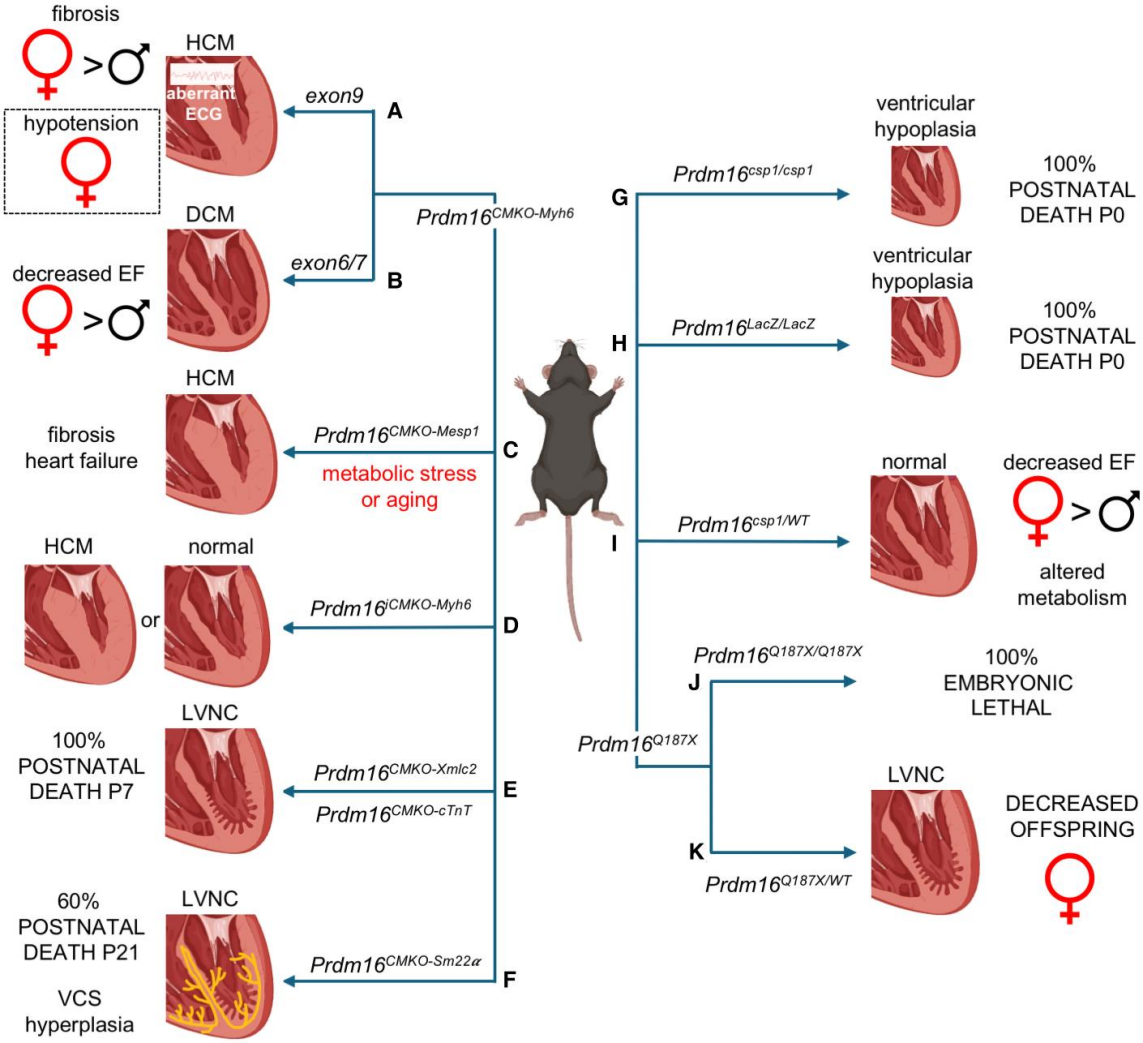
Heterogeneity in preclinical mouse models with myocardial PRDM16 deficiency. (*A*) Cardiomyocyte (CM)-specific deletion of *Prdm16* using the *Myh6* Cre-driver induced hypertrophic cardiomyopathy (HCM) and hypotension, respectively more pronounced or exclusively present in female mice. (*B*) Using the same Cre-driver, but a different *Prdm16^fl/fl^* mouse model, with *LoxP* sites flanking *exon 6/7*, dilated cardiomyopathy (DCM) was observed with reduced ejection fraction (EF), again more pronounced in female mice. (*C*) In a CM-specific KO model using *Mesp1* as a Cre-driver, HCM was seen only upon metabolic stress or ageing. (*D*) An adult inducible (using tamoxifen) CM-specific Cre-recombinase model based on *Myh6* resulted in HCM in one study but no functional abnormalities in another report. (*E*) In a CM-specific mouse model using *Xmlc2* or *cTnT* as Cre-drivers, postnatal mortality was seen in the complete offspring by 7 days of age (P7). Embryonic hearts showed a left ventricular non-compaction (LVNC) phenotype. (*F*) CM-specific deletion of *Prdm16* during cardiac development using a *Sm22α*-driven Cre-recombinase resulted in contractile dysfunction, hyperplasia of the ventricular conduction system (VCS), and hence abnormal electrophysiology of the postnatal heart, resulting in premature death by P21. (*G*, *H*) Ubiquitous homozygous *Prdm16*-KO mouse models are lethal postnatally and feature left ventricular hypoplasia. (*I*) Ubiquitous heterozygous deletion of *Prdm16* (*csp1* mutant) showed normal heart morphology but reduced EF and an altered metabolism more pronounced in females. (*J*, *K*) Overexpression of the nonsense variant Q187X was embryonic lethal in a homozygous setting and displayed LVNC in a heterozygous setting. Heterozygous females were slightly underrepresented in the offspring. The figure was designed in BioRender.

**Figure 4:**
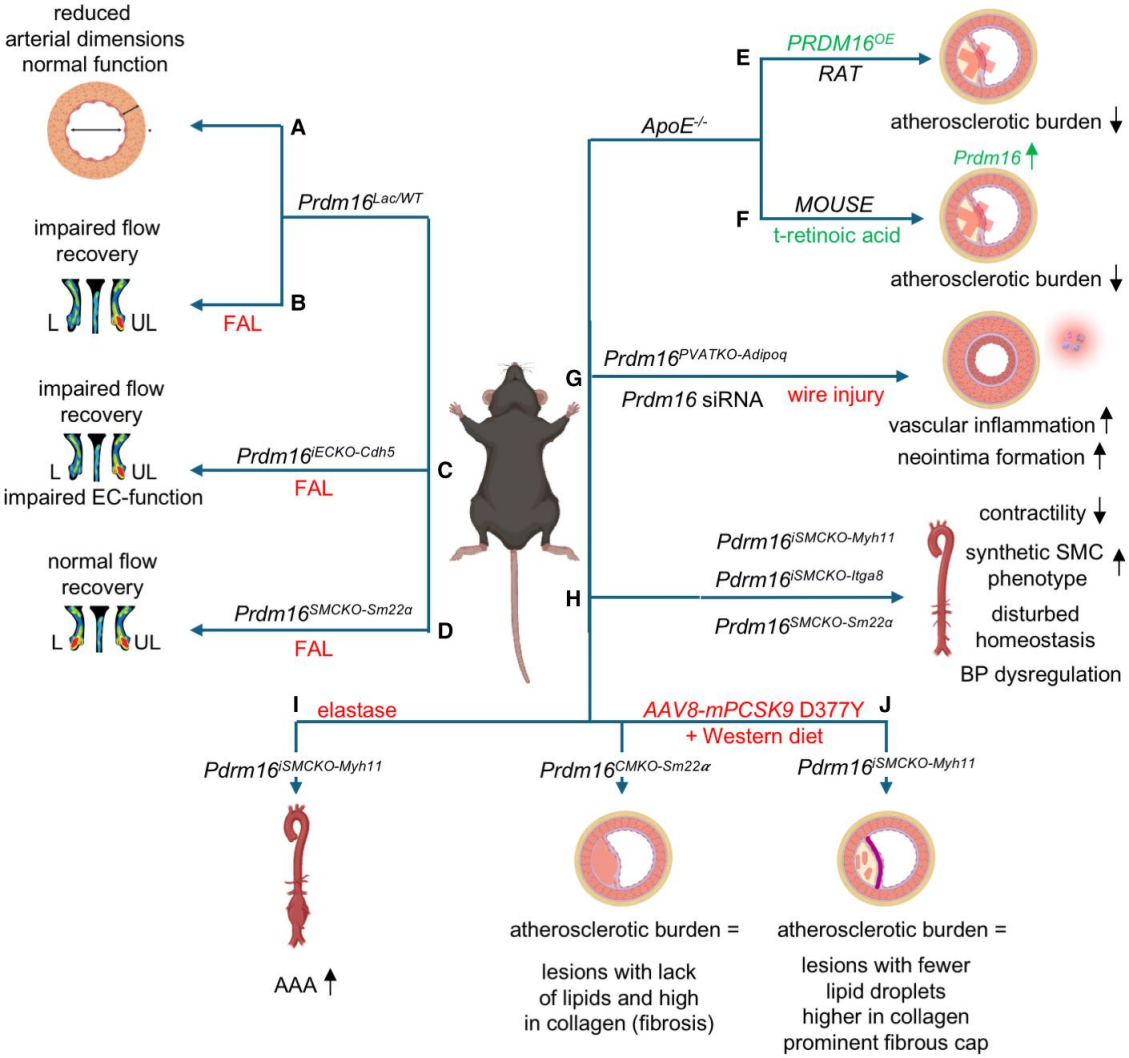
Preclinical mouse models with PRDM16 deficiency in the vascular system. (*A*) Ubiquitous heterozygous PRDM16-deficient mice demonstrated reduced arterial dimensions compared to *wild-type* (*WT*) littermates. (*B*) In the same model, after induction of femoral artery ligation (FAL) impaired flow recovery was seen. L: ligated; UL: unligated. (*C*) In an endothelial cell (EC)-specific knockout (KO) model using the inducible *Cdh5* Cre-driver, this impaired flow recovery after FAL was recapitulated. (*D*) The same study showed that the impairment in blood flow recovery following FAL was not observed in mice with smooth muscle cell (SMC)-*Prdm16* deletion. (*E*) PRDM16 overexpression (*PRDM16^OE^*) in *ApoE^−/−^* rats led to decreased atherosclerotic plaque formation. (*F*) *Prdm16* expression was upregulated in perivascular adipose tissue of *ApoE^−/−^* mice, showing reduced atherosclerosis following all trans (t)-retinoic acid treatment. (*G*) Adipose-specific deletion of *Prdm16* using the *Adipoq* Cre-driver or by locally silencing *Prdm16* expression via *Prdm16* siRNA resulted in exacerbated vascular inflammation and neointima formation following endovascular wire injury. (*H*) SMC-specific deletion of *Prdm16* using *Myh11*-, *Itga8- or Sm22α-Cre* models showed reduced contractility, disrupted SMC homeostasis, and dysregulated blood pressure (BP). (*I*) Using the *Myh11-Cre* mouse model, elastase-induced abdominal aortic aneurysms (AAAs) were aggravated compared to challenged *WT* controls. (*J*) SMC-*PRDM16* deletion using the *Myh11-Cre* or the *Sm22α-Cre* mouse model did not affect atherosclerotic burden (upon adeno-associated viral (AAV) transfer of a gain-of-function proprotein convertase subtilisin/kexin type 9 (PCSK9) mutant and exposure to Western diet) but altered atherosclerotic plaque composition. The figure was designed in BioRender.

Using transgenic experimental models, multiple research groups, including us, have discovered that PRDM16 is essential for cardiovascular development and preserving cardiovascular function in physiological and pathological conditions. Below, we provide an overview of preclinical studies investigating (loss of) PRDM16 function and the underlying (molecular) mechanisms in different models of CVD (*Figures 3* and *4*).

### 3.1 The role of PRDM16 in cardiac function and disease: experimental models

Arndt and colleagues were the first to study the role of PRDM16 in cardiac development and function after identifying a dominant negative mutation in *PRDM16* (*PRDM16^K702Q^)* in patients with 1p36 deletion syndrome suffering from dilated cardiomyopathy (DCM) and left ventricular non-compaction (LVNC), the latter characterised by a thin compact outer myocardial wall and a persistent luminal trabecular layer.^31^ They further investigated the cardiac function of PRDM16 in zebrafish and found that both knockdown of *prdm16* or CM-specific overexpression of human PRDM16^K702Q^ significantly reduced cardiac output and stroke volume 72 hours-post-fertilisation (hpf). Moreover, total CM numbers were significantly decreased compared to *wild-type* (*WT*) controls, due to a significant decrease in CM proliferation at 28, 48, and 72 hpf. Optical mapping revealed abnormal cardiac conduction in zebrafish embryonic hearts with perturbed prdm16 signalling, suggesting partial cellular uncoupling which is often reciprocally related to proliferation. Consistent with these findings, other research groups have now suggested that impaired proliferation of the compact myocardial wall could result in LVNC.^39,52^

Bjork *et al*. briefly alluded to the role of PRDM16 during cardiac development in their seminal paper in 2010–2013, years before Arndt *et al.—*by reporting ventricular hypoplasia in a ubiquitous PRDM16-deficient mouse model.^12^ Yet, the first conditional (i.e. CM-specific) *Prdm16* knockout (KO) mouse strains were generated only 10 years later. By intercrossing diverse CM-specific Cre-drivers with the *Prdm16^lox/lox^* mouse line harbouring floxed *exon 9* alleles,^53^  *Prdm16* was deleted specifically in CMs (from here on referred to as ‘*Prdm16^CMKO-Cre-driver^’*). Using different Cre deleter lines, we and others induced CM-specific *Prdm16* KO in a spatiotemporal fashion, to study the role of PRDM16 during cardiac development or within the adult heart. These studies have however led to remarkably diverse phenotypic outcomes (*Figure 3*).

The first *Prdm16^CMKO^* mouse model featured the constitutive *myosin heavy chain* (*Myh)6*-Cre-driver (*Figure 3A*), which is reported to target 90% of all CMs at the onset of activity (i.e. at E8.0).^54,55^  *Prdm16^CMKO-Myh6^* mice lived until adulthood but featured an increased QRS duration and QTc interval in both males and females, likely attributable to perturbed ion channel homeostasis. Moreover, *Prdm16^CMKO-Myh6^* mice displayed cardiac hypertrophy and enhanced interstitial fibrosis compared to control littermates.^54^ An independent research group later reported that Myh6-driven *Prdm16* deletion additionally leads to DCM, although they used a different *Prdm16^lox/lox^* mouse model with the *LoxP* sites flanking *exon 6/7* (*Figure 3B*).^56^ Both studies showed a more pronounced cardiac phenotype in female *Prdm16^CMKO-Myh6^* mice, as observed in patients carrying pathogenic *PRDM16* mutations.^56^ Intriguingly, female *Prdm16^CMKO-Myh6^* mice, in addition to cardiac hypertrophy, also featured hypotension due to increased expression of inducible nitric oxide (NO) synthase, further highlighting sex-dependent cardiovascular outcomes in PRDM16-deficient mice (*Figure 3A*).^57^

Surprisingly, deletion of *Prdm16* at the earliest time point of cardiac development, using a constitutive *Mesp1-Cre* driver (*Prdm16^CMKO-Mesp1^*; *Figure 3C*), only resulted in cardiac hypertrophy in late adulthood. The underlying mechanism involved the recruitment of EHMTs by PRDM16 to form a triple complex with the DNA-binding and pro-hypertrophic TF MYC, thereby condensing the chromatin of hypertrophy genes and reducing their expression.^32^ Hence, this study evidenced the epigenetic function of PRDM16 in the heart, as previously shown in other tissues (*Figure 1D* and *E*).^18,23,58^ Additionally, *Prdm16^CMKO-Mesp1^* hearts showed increased fibrosis, mitochondrial dysfunction, and impaired metabolic flexibility, as young (3-months old) *Prdm16^CMKO-Mesp1^* mice developed heart failure in response to metabolic stress (i.e. high-fat diet).^32^ When deleting *Prdm16* at the age of 8 weeks using a tamoxifen-inducible *MerCreMer-Myh6* driver (i.e. *Prdm16^iCMKO-Myh6^*), Cibi *et al*. equally observed cardiac hypertrophy and reduced expression of mitochondrial genes 6 weeks later (*Figure 3D*).^32^

A more severe, early-onset phenotype was observed by Wu *et al.* using an *Xmlc2-Cre* deleter line,^39^ which shows highly efficient Cre-recombinase activity in CMs as early as E7.5 (*Figure 3E*). In contrast to previous models, the *Prdm16^CMKO-Xmlc2^* model resulted in embryonic biventricular non-compaction and left ventricular-specific dilatation, mirroring the phenotypic characteristics of patients with *PRDM16* mutations. Moreover, these mice displayed progressive cardiac dysfunction resulting in death before the postnatal day (P)7, which the authors also observed in a second mouse model using *cTnT-Cre* deleter mice (i.e. *Prdm16^CMKO-cTnT^*).^39^ However, deletion of *Prdm16* in adult CMs (i.e. *Prdm16^iCMKO-Myh6^*) did not—unlike in the report by Cibi *et al.*—result in obvious cardiac defects in their hands, indicating that PRDM16 is indispensable for proper cardiac development but not essential for cardiac function during adulthood, in line with its very low expression in adult myocardium (*Figure 3D*).^32,39^

Mechanistically, PRDM16 represses the expression of trabecular genes, while promoting compact gene expression, in line with its enrichment in the compact myocardium during development. As a result, loss of PRDM16 in CMs leads to an identity shift of compact myocardial cells towards trabecular CMs, resulting in a thin outer compact myocardial layer and an excessive luminal trabecular meshwork. This dual role—previously described in PRDM16-mediated brown vs. white adipose specification—is attributable to (in)direct binding of PRDM16 to both promoters and distant enhancer regions. Intriguingly, PRDM16 affected gene expression mostly in the left ventricle despite its equal expression in left and right ventricles, due to its cooperative effect with TFs which have a left ventricular-skewed expression pattern, including HAND1 and TBX5.^39^

Recently, we found an additional role for PRDM16 in cardiac development using *Sm22α-Cre* deleter mice.^33^  *Sm22α* is expressed in CMs between E8.0 and E12.5,^59^ a crucial time window for ventricular wall development. In analogy to previous reports^39,54,60^ and in line with the timing of our conditional KO model, we showed that PRDM16 deficiency in *Prdm16^CMKO-Sm22α^* leads to a reduced compact myocardial wall resembling LVNC, associated with severe cardiac dysfunction, conduction abnormalities, and premature death in about 60% of the Cre^+^ offspring (*Figure 3F*). In addition to the compact-to-trabecular lineage shift observed by others at E13.5,^39^ we employed a multi-omics strategy at single-cell resolution to demonstrate a switch from ventricular working CMs to a more atrial and ventricular conduction-like molecular profile in P7 *Prdm16^CMKO-Sm22α^* mice compared to *WT* littermates. Additional gene regulatory network analysis showed that PRDM16 favours ventricular working CM identity by opposing the activity of master regulators of ventricular conduction and atrial fate, e.g. TBX5, TBX3, and GATA5. Together, these perturbations in CM lineage specification resulted in ventricular conduction system hyperplasia, which is a remarkable finding, as abnormalities in the ventricular conduction system are most typically manifested as hypoplasia.^61^

Importantly, these studies—which have unravelled key functions of PRDM16 at different time points of cardiac development—do not reflect patients with PRDM16-related cardiomyopathy that are heterozygous for the *PRDM16* mutation. Although Bjork *et al.* found that homozygous offspring of the first two germline constitutive PRDM16-deficient mouse strains generated (i.e. *Prdm16^csp1/WT^* and *Prdm16^LacZ/WT^*) dies shortly after birth due to respiratory defects and displays a hypoplastic left ventricle (*Figure 3G* and *H*),^12^ cardiovascular defects in surviving heterozygous offspring were not reported. Recently, Kühnisch and colleagues described metabolic alterations, transcriptional perturbations, and mildly reduced cardiac performance in *Prdm16^csp1/WT^* mice, especially in females (*Figure 3I*).^42^ Shortly after this report, Sun and colleagues created knock-in mice harbouring the nonsense *Prdm16^Q187X^* variant which causes reduced PRDM16 expression by nonsense-mediated decay and was associated with cardiomyopathy in patients.^60^ Homozygous *Prdm16^Q187X/Q187X^* mice showed an underdeveloped compact myocardium and died—unlike the postnatal death at P7 reported by Wu *et al.*^39^—before birth (*Figure 3J*).^60^ Heterozygous *Prdm16^Q187X/WT^* mice showed impaired myocardial development leading to non-compaction in adult mice (*Figure 3K*). Although *Prdm16^Q187X/WT^* × *Prdm16^WT/WT^* offspring showed a Mendelian distribution, there was a slightly lower-than expected proportion of female *Prdm16^Q187X/WT^* mice born (*Figure 3K*).^60^ These findings once again highlight sexual dimorphism in PRDM16-related cardiomyopathy in preclinical animal models.

Overall, these studies evidenced the indispensable role of PRDM16 in cardiac development and function, and the variety of cardiac phenotypes seen in the distinct models likely reflects the broad clinical landscape seen in *PRDM16*-related cardiomyopathy patients (*Table 1*). This phenotypic heterogeneity is also seen for other cardiomyopathy-related genes (e.g. *MYH7*) where different mutations in the same gene lead to a wide spectrum of cardiomyopathy phenotypes like DCM, hypertrophic cardiomyopathy (HCM), and LVNC.^74^ The differences amongst Cre-driver KO models are presumably the consequence of variable temporal and spatial promoter—and hence Cre-recombinase—activities. For instance, while MESP1 is known for its actions in multipotent cardiogenic progenitors, at least 30% of CMs do not originate from MESP1^+^ progenitors.^75,76^ Furthermore, MESP1 is expressed within a limited time frame (E6.0-7.0) that does not coincide with the earliest reported cardiac expression of PRDM16 (at E10.5), which could result in low recombination efficiency and a rather mild phenotype manifested only in adulthood.^32,77^ Likewise, the predominant expression of MYH6 in atria at E8.0, and its declining expression in ventricles from E10.5 until birth (which coincides with the expression of PRDM16 in the ventricles) may limit recombination activity and hence obscure the important role of PRDM16 during cardiac development.^54,56,57,78^ On the other hand, the *Xmlc2-*, *cTnT2*-, and *Sm22α-*Cre-driver lines are broadly active from E7.5-8.0 onwards, resulting in a higher recombination efficiency and a dramatic early-onset phenotype.^33,39^ Although we speculate that these different Cre-drivers are the culprit for the variety of cardiac phenotypes, sex differences, and genetic backgrounds are likely contributing factors to this phenotypic heterogeneity.

**Table 1:** Genetic *PRDM16* variants in patients with cardiac disease

Diagnosis	Variant		Varsome^a^	Year	Type of study	Patient population	Reference
ARVC	*PRDM16 c.2666C > T p.P889L*	*rs201814961*	VUS	2022	Retrospective single-centre cohort study	German population (*n* = 4)	^62^
	*PRDM16 c.776C > T p.A259V*	*rs769041652*	VUS				
	*PRDM16 c.2056A > G p.T686A*	*rs575376153*	LB				
CMP	1p36 deletion			2023	Retrospective multi-centre cohort study	American population (*n* = 71)	^56^
DCM	*PRDM16 c.872C > T [p.Pro291Leu]*	*rs397514744*	VUS/P	2013		European population (*n* = 131)	^31 b^
	*PRDM16 c.2660T > C [p.Leu887Pro]*	*rs202115331*	P				
	*PRDM16 c.3301G > A [p.Val1101Met]*	*rs201654872*	B				
	*PRDM16 c.811G > A (p.Glu271Lys)*	*rs200052869*	VUS				
	*PRDM16 c.2187C > G p.F729L*	*rs200109766*	LB	2022	Retrospective single-centre cohort study	German population (*n* = 54)	^62^
	*PRDM16 c.2603 + 6C > T*	*rs374549827*	LB				
Foetal LVNC	PRDM16 c.1057C > T;p.(Gln353*)	*NA*	NA	2020	Case report	Not available (*n* = 1)	^63^
HCM	*PRDM16 c.668G > T p.G223V*	*rs1202885373*	NA	2022	Retrospective single-centre cohort study	German population (*n* = 47)	^62^
	*PRDM16 c.2296G > A p.G766S*	*rs199998420*	LB				
LVNC	1p36 deletion			2008	Prospective cohort study	Italian population (*n* = 60)	^64^
	1.52MB deletion			2011	Case report	Not available (*n* = 2)	^65^
	*PRDM16 c.2104A > T [p.Lys702∗]*	*rs397514742*	P	2013	Retrospective multi-centre cohort study	European population (*n* = 75)	^31,66^
	*PRDM16 c.1573dupC [p.Arg525Profs∗79]*	*rs886041395*	P				
	*PRDM16 c.2447A > G [p.Asn816Ser]*	*rs397514743*	VUS				
	PRDM16 c.56delA p.(Asn19Ilefs*114)	*NA*	NA	2018		Dutch population (*n* = 327)	^66,67^
	*PRDM16 c.676 + 1G > A*	*NA*	NA				
	PRDM16 c.2848C > T p.(Arg950*)	*rs1294136105*	VUS				
	*PRDM16 c.1040C > T p.(Thr347Met)*	*rs746868217*	VUS				
	*PRDM16 c.1633G > A p.(Ala545Thr)*	*rs76326870*	NA				
	*PRDM16 c.1840G > A p.(Asp614Asn)*	*rs764738323*	VUS				
	*PRDM16 c.567T > G p.(Ser189Arg)*	*rs368081666*	VUS				
	*PRDM16 c.2134C > T p.Gln712Ter*	*NA*	NA		Not available	Not available	ClinVar database^c^
	*PRDM16 c.3091G > A p.Glu1031Lys*	*rs373883664*	VUS	2019	Prospective multi-centre cohort study	French population (*n* = 95)	^68^
	*PRDM16 c.561G > C p.Gln187His*	*rs752812879*	VUS				
	*PRDM16 c.534C > A p.Cys178Ter*	*rs1643176372*	P	2020	Not available	Not available	ClinVar database^d^
	*PRDM16 c.213del p.Val72fs*	*rs1569798933*	VUS/P				ClinVar database^e^
	*PRDM16 c.2809_2810dupCC p.Thr938GlnfsX34*	*rs876657961*	VUS	2021			^66^ ClinVar database^b,f^
	*PRDM16 c.2434del p.Arg812fs*	*NA*	LP				ClinVar database^g^
	*PRDM16 c.3271C > T p.Arg1091Ter*	*NA*	LP	2022			ClinVar database^h^
	PRDM16 c.559C > T p.Gln187*	*NA*	P	2023	Retrospective study	American population (*n* = 2)	^60^
	*PRDM16 c.676 + 2T > C*	*NA*	NA				
	*PRDM16 c.3142del p.Leu1048fs*	*NA*	LP		Not available	Not available	ClinVar database^d^
	*PRDM16 c.2595dup p.lle886fs*	*rs1643715509*	P	2024			ClinVar database^i^
	*PRDM16 c.1110C > A p.D370E*	*rs777550737*	VUS	2022	Retrospective single-centre cohort study	German population (*n* = 119)	^62^
	PRDM16 c.1627C > T p.Q543*	*rs2100662912*	P				
	*PRDM16 c.1885G > C p.V629L*	*NA*	NA				
	*PRDM16 c.2691 + 5G > A*	*rs375994227*	VUS				
Myocarditis	*PRDM16 c.115G > A p.E39K*	*rs775285788*	VUS			German population (*n*= 54)	
Paediatric DCM	PRDM16 c.1047dupC p.S350fs*48	*rs1643547095*	VUS	2018	Pro- and retrospective single-centre study	American population (*n* = 21)	^69^
Paediatric LVNC	PRDM16 c.1286_1289delinsTTGCACTT p.(Gly429Valfs*176	*NA*	NA	2022	Prospective single-centre cohort study	Polish population (*n* = 31)	^70^
	PRDM16 c.1336G > T p.(Glu446*)	*NA*	NA				
QRS alteration	*PRDM16 c.439-46177G > A*	*rs2483280*	NA	2014	GWAS	Asian population (*n* = 14 100)	^71^
	*PRDM16 c.1154-1894T > A*	*rs335206*	NA				
RCM	*PRDM16 c.2372G > A p.G791D*	*rs1569732779*	VUS	2022	Retrospective single-centre cohort study	German population (*n* = 8)	^62^
WPW syndrome	*PRDM16 c.2666C > T:p* (Pro889Leu)	*rs201814961*	VUS	2020		American population (*n* = 305)	^72^
	*PRDM16 c.2855C > T:p* (Thr925Met)	*rs749180764*	VUS				

ARVC, arrhythmogenic right ventricular cardiomyopathy; CMP, cardiomyopathy; DCM, dilated cardiomyopathy; LVNC, left ventricular non-compaction cardiomyopathy; HCM, hypertrophic cardiomyopathy; RCM, restrictive cardiomyopathy; LOF, loss of function; SNP, single nucleotide polymorphism; GWAS, genome-wide association study; WPW, Wolff-Parkinson-White; (L)P, (likely)pathogenic variant; LB, likely benign variant; VUS, variant of unknown significance; NA, not applicable/available.

^a^Varsome assessment^73^ of pathogenicity; if more than 1 designation is mentioned, then this reflects conflicting assessments by different studies.

^b^Original publication in case of multiple references.

^c^Illumina Laboratory Services, Illumina.

^d^Baylor Genetics.

^e^Rady Children's Institute for Genomic Medicine, Rady Children's Hospital San Diego.

^f^Laboratory for Molecular Medicine, Mass General Brigham Personalized Medicine.

^g^MGZ Medical Genetics Center München.

^h^Victorian Clinical Genetics Services, Murdoch Children’s Research Institute.

^i^Ambry Genetics.

### 3.2 The role of PRDM16 in vascular function and disease: experimental models

As mentioned before, PRDM16 has been identified as a key transcriptional regulator to induce and maintain arterial endothelial identity *in vivo* and *in vitro* and is involved in developmental and early postnatal angiogenesis and arteriogenesis.^43,44,49^ This crucial role in arterial ECs during development and its persistent expression in the adult vasculature has instigated research into the role of PRDM16 in adult vascular function and disease. We demonstrated that heterozygous PRDM16-deficient mice (*Prdm16^LacZ/WT^*) had slightly smaller inner diameters of the DA and femoral artery (FA) compared to their *WT* littermates.^28^ Reduced arterial calibres were also associated with decreased SMC-coating (*Figure 4A*), in line with the previously mentioned findings from Thompson *et al*. (in Section 2.2).^44^

Nevertheless, baseline vasomotor function and biomechanical properties of both the DA and FA (and accordingly blood pressure) were not affected by PRDM16 heterozygous deficiency under physiological conditions. In contrast, PRDM16 was found to be an essential TF to maintain normal arterial EC function under pathological conditions, such as during peripheral artery disease (PAD) induced by FA ligation.^28^ Indeed, both heterozygous and EC-specific *Prdm16* KO mice displayed impaired arterial blood flow recovery compared to their *WT* littermates (*Figure 4B*), a phenotype not observed after conditional deletion of *Prdm16* in SMCs (using the 40% fraction of *Prdm16^SMCKO-Sm22α^* mice surviving until adulthood; *Figure 4C* and *D*).^28^ Intriguingly, this flow impairment was not related to defects in angiogenesis or arteriogenesis, since capillary or collateral growth were not impaired by PRDM16 loss.^28^ Rather, endothelial *Prdm16* deletion compromised flow recovery by priming the arterial endothelium for ischaemia-induced dysfunction, as evidenced by reduced NO bioavailability and aberrant calcium (Ca^2+^) homeostasis leading to impaired EC-dependent relaxation of the FA.^28^ Importantly, these phenotypes—observed in mice in which *Prdm16* was deleted from P1 onwards—were milder and delayed in mice with short-term deletion (i.e. from 2 weeks before FA ligation), suggesting that loss of PRDM16 results in a gradually increased susceptibility to endothelial dysfunction.^28^ However, in the absence of chromatin immunoprecipitation followed by sequencing (ChIP-seq) data in arterial ECs, the mode of action through which PRDM16 affects arteriovenous specification and arterial EC function before and after birth remains to be determined.

Importantly, several preclinical studies have suggested a role for PRDM16 in the initiation/progression of arterial-restricted diseases, such as atherosclerosis, coronary artery disease (CAD), and abdominal aortic aneurysms (AAAs). Despite the prominent role of endothelial dysfunction in these vascular disorders, the majority of these studies focus on PRDM16's role in other compartments of the vascular wall where PRDM16 is more prominently expressed, including the SMC layer^79^ and the perivascular adipose tissue (PVAT).^80^ The latter undergoes browning in response to vascular injury, with beneficial effects on vascular inflammation and pathological vascular remodelling. Accordingly, it was shown that adenoviral-mediated overexpression of PRDM16 in atheroprone (*ApoE^−/−^*) rats led to the inhibition of atherosclerotic plaque formation and was accompanied by the conversion of WAT into BAT surrounding the abdominal aorta (*Figure 4E*).^81^ However, the beneficial effects of PRDM16 in this model could extend beyond PVAT, as PRDM16 overexpression was driven by a CMV promoter, and no *in situ* documentation of the cells effectively targeted by this approach was provided.^81^ Nonetheless, decreased atherosclerotic burden after trans-retinoic acid treatment of *ApoE^−/−^* mice correlated with increased *Prdm16* expression in PVAT (*Figure 4F*)^82^ and adipose-specific deletion or local siRNA-mediated silencing of *Prdm16* suppressed beiging of PVAT and exacerbated inflammation and neointima formation after endovascular wire injury (*Figure 4G*).^83^ The reciprocal cross-talk between adipose tissue and the cardiovascular system whereby one affects the maintenance and function of the other by secretion of signals (e.g. adipokines and angiocrine molecules) is well-known.^84^ PRDM16, being expressed on both sides may therefore play an intriguing role in this cross-talk.

Recently, an important role for PRDM16 in vascular SMC homeostasis and function was identified. SMC-specific *Prdm16* deletion (driven by the *Myh11-Cre* deleter line) induced the expression of genes involved in extracellular matrix (ECM) remodelling and inflammation in the abdominal aorta, indicative of an enhanced susceptibility for vascular disorders.^85^ Correspondingly, Dong *et al.* demonstrated that loss of PRDM16 in SMCs resulted in the upregulation of genes implicated in SMC phenotypic switching and the concomitant downregulation of genes associated with SMC differentiation and contraction.^37^ This switch towards a synthetic SMC phenotype upon PRDM16 loss was recently confirmed by two other reports (*Figure 4H*).^79,80^ Hence, like in ECs and adipocytes, PRDM16 plays a bifunctional role in regulating SMC homeostasis by suppressing and promoting alternative cell fates. Accordingly, adult mice deficient for PRDM16 in their SMCs (either by using the *Myh11-, Itga8-* or *Sm22α-Cre* deleter lines) were shown to have reduced contraction of their resistance arteries and lower blood pressure and disturbed blood pressure circadian variation, the latter associated with aberrant expression of PRDM16 target genes such as adrenoreceptor *Adra1d* and clock gene *Npas2* (*Figure 4H*).^79,80^

In an elastase-induced AAA mouse model, SMC-specific *Prdm16* KO mice showed aggravated AAA formation compared to their *WT* littermates (*Figure 4I*), while knockdown of *Prdm16* in cultured SMCs induced inflammation and apoptosis.^85^ Mechanistically, PRDM16 drives expression of ECM components, while suppressing *Adam12*, encoding an ECM-remodelling proteinase. These findings are likely relevant for human disease, as reduced *Prdm16/PRDM16* expression and increased levels of *Adam12/ADAM12* were found in the aortic tissues of both mouse and human AAAs.^85^ Moreover, *Adam12* was required for the pro-apoptotic effects of PRDM16 deficiency in SMCs, suggesting that the association between PRDM16 and ADAM12 may play a key role in AAA formation.^85^

Recently, Tan *et al*. documented that loss of PRDM16 in SMCs of *Prdm16^SMCKO-Sm22α^* or *Prdm16^iSMCKO-Myh11^* mice results in maladaptive SMC phenotypic switching in a mouse model of atherosclerosis,^79^ consistent with the reduced PRDM16 mRNA or protein levels in SMCs of atherosclerotic vs. healthy human and mouse arteries^37,79^ and with the identification of PRDM16 as a central determinant of SMC-dependent risk for atherosclerosis.^36^ While the switch of PRDM16-deficient SMCs to a synthetic and proliferative phenotype did not alter atherosclerotic burden, it resulted in prominent changes in plaque composition, i.e. an increase in collagen content and a decrease in lipid-laden cells in both conditional models and the formation of thick fibrous caps upon the acute loss of SMC-PRDM16 in the inducible *Prdm16^iSMCKO-Myh11^* model (*Figure 4J*). Mechanistically, PRDM16 represses a synthetic phenotype in SMCs by binding to chromatin and decreasing activating histone marks at synthetic genes.^79^

Together, these studies suggest that PRDM16 protects against arterial disorders via its effect on ECs, PVAT cells, and SMCs, and that loss of PRDM16 invariably leads to transcriptional changes with associated pathogenic, maladaptive cell states that contribute to disease progression. As reduced *Prdm16* expression has been observed in atherosclerotic^37^ and AAA lesions^85^ in both human and mouse aortic tissues, it is conceivable that reduced PRDM16 levels are not just a mere consequence of arterial disease, but rather a key aspect of their pathogenesis. Moreover, as PRDM16 is expressed in multiple cell types involved in arterial disease, reduced PRDM16 levels likely perturb the cross-talk between ECs, SMCs, and PVAT cells, with detrimental consequences for arterial health and function. These findings further highlight PRDM16 as an important drug target to treat arterial disorders such as atherosclerosis and aortic aneurysms.

## 4. PRDM16 and TGF-β signalling have a complex relationship in the cardiovascular system

PRDM16 can bind SMAD proteins and is known to inhibit the TGF-β pathway during embryonic development in the skeletal system and during cancer.^8,10^ Likewise, numerous studies have indicated that the cardiac phenotypes downstream of perturbed PRDM16 signalling are—at least partly—attributable to alterations in TGF-β signalling. Arndt *et al*. showed increased TGF-β signalling in *prdm16*-deficient zebrafish hearts, likely responsible for the observed brake on CM proliferation^31^ that was later proposed to be the culprit for the aberrant myocardial wall development seen during LVNC.^39,52^ The inhibitory role of PRDM16 on TGF-β signalling was echoed by the binding of PRDM16 to the promoter and reduced expression of *TGFB3* in H9c2 cardiomyoblasts.^60^ Similarly, the latter report found impaired proliferation and increased apoptosis in *PRDM16^Q187X^* iPSC-CMs, which was associated with increased expression of genes implicated in TGF-β signalling.^60^ Accordingly, an earlier study in iPSC-CMs carrying a frameshift mutation in *PRDM16 exon 9* showed impaired CM proliferation and upregulated TGF-β target genes.^86^ Likewise, the upregulation of TGF-β1, TGF-2, and TGF-3 ligands was postulated as a possible cause for the hypertrophy and fibrosis in *Prdm16^CMKO-Myh6^* mice, although the QRS abnormalities could not be reversed by inhibition of TGF-β signalling.^54^ Intriguingly, impaired myocardial development in *Prdm16^Q187X/WT^* mice correlated with reduced rather than increased TGF-β signalling,^60^ while adult *Prdm16^Q187X/WT^* hearts displayed increased TGF-β signalling with corresponding pathogenic remodelling.^60^ Together, these studies demonstrate that PRDM16 has a complex time- and context-dependent relationship with TGF-β signalling in the CM lineage. This complex interference of PRDM16 with TGF-β signalling may also underlie the cell-type dependent effects it has on proliferation. Indeed, while PRDM16 promotes proliferation in the myocardial wall by repressing the anti-proliferative activity of TGF-β, it inhibits proliferation and regulates quiescence of HSCs and NSCs, as mentioned above (in Section 1).^13,15–17^ For the latter, the induction of quiescence was recently shown to involve the BMP-phosphoSMAD1/5/8 axis.^17^ Hence, depending on the presence of specific co-factors, PRDM16 is able to control cell cycle dynamics in a bidirectional fashion.

Interestingly, like in CMs, *Tgfb2* was identified to be one of the major targets underlying PRDM16's function in regulating SMC homeostasis. Expression of the *Tgfb2* gene was increased upon PRDM16 loss in SMCs and *Tgfb2* harboured PRDM16 ChIP-seq peaks within its promoter region, implicating that *Tgfb2* is a direct target of PRDM16.^37^ As TGF-β is a major regulator of SMC function and aberrant TGF-β signalling negatively influences SMC homeostasis,^87^ it is tempting to speculate that the function of PRDM16 within SMC might also be (largely) mediated through its effect on TGF-β expression or signalling. However, more research is needed to identify the exact mechanism, functions, and targets of PRDM16 in the healthy and diseased vasculature. The latter is particularly true for its role in ECs where the lack of ChIP-seq and assay for transposase-accessible chromatin followed by sequencing (ATAC-seq) data has prevented mapping out PRDM16's direct and indirect targets.

## 5. Clinical evidence for the role of PRDM16 in CVDs

### 5.1 The role of PRDM16 in cardiac disease: clinical studies

Numerous genetic studies have linked mutations in different regions of the *PRMD16* gene to cardiomyopathies in patients, many of which are estimated as pathogenic (*Table 1*; *Figure 1A*). Triggered by the reported ventricular hypoplasia in homozygous *Prdm16* KO mice,^65^ Gajecka and colleagues were one of the first to suggest a role for *PRDM16* in LVNC observed in patients with chromosome 1p36 deletion syndrome (a DNA region that encompasses the *PRDM16* locus). This syndrome yearly affects approximately 1:5000 newborns and often coincides with cardiac defects,^88^ including phenotypic characteristics of LVNC in many patients.^64^ Arndt *et al.* demonstrated that 1p36 syndrome-associated LVNC was linked to the deletion of *exons 4–17* of *PRDM16*, and further detected *PRDM16* mutations in an independent cohort of non-syndromic LVNC patients.^31^ A retrospective, Dutch multi-centre study for LVNC further identified 3 pathogenic mutations in *PRDM16*, including one *de novo* mutation,^67^ while Delplancq *et al.* attributed a foetal case of severe cardiomegaly and endocardial fibro-elastosis with associated LVNC to a novel heterozygous nonsense *PRDM16* variant in combination with missense variants in *TTN*.^63^ Two novel, unrelated probands with paediatric LVNC and the onset of heart failure were described with loss-of-function *PRDM16* variants, one being the abovementioned *PRDM16^Q187X^* variant in *exon 4* of the *PRDM16* gene, another being a splice variant of *PRDM16 exon 5*.^60^ Similarly, a single-centre paediatric study identified two *de novo* pathogenic variants in the *PRDM16* gene, which were associated with childhood LVNC.^70^ A large-scale assessment of the genetic architecture of LVNC, performed in 6 different study cohorts, reported both known^31,67^ and *de novo PRDM16* variants and found that, unlike mutations in most genes, including those encoding sarcomeric proteins, *PRDM16* variants exclusively associated with LVNC but not HCM and DCM.^66^

Nonetheless, it has become increasingly clear from other study cohorts that impaired PRDM16 signalling may not only predispose to LVNC, but also to other types of cardiomyopathy. In addition to the 1p36 deletion-associated LVNC in the patient cohort of Arndt *et al.*, cardiac biopsies from DCM patients were analysed, in which 4 new non-synonymous *PRDM16* variants were discovered, which were not observed in healthy controls.^31^ Around the same time, Long *et al.* associated a *de novo* frameshift mutation in *PRDM16* with paediatric DCM.^69^ Exome sequencing on families with Wolff-Parkinson-White syndrome, a relatively common arrhythmia, revealed *de novo* variants in genes associated with arrhythmia and cardiomyopathy, including *PRDM16.*^72^ These findings are consistent with limited reports that link PRDM16 to arrhythmias or QRS irregularities, including a genome-wide association study on electrocardiographic traits performed in an Asian population.^71^ Finally, a systematic analysis of 285 paediatric and adult cardiomyopathy patients revealed 16 heterozygous *PRDM16* variants of interest, of which 3 were classified as pathogenic. Interestingly, the phenotypes of these patients did not only display LVNC and DCM, but also included HCM, restrictive cardiomyopathy, and arrhythmogenic right ventricular cardiomyopathy.^62^

Together, these studies have unequivocally demonstrated the link between heterozygous *PRDM16* mutations and phenotypically diverse cardiomyopathies. While LVNC was the most common clinical feature, the cardiac phenotypes observed due to PRDM16 deficiency span the entire spectrum of cardiomyopathies, including conduction abnormalities. Hence, genetic testing, coupled with research aimed at a better understanding of the molecular underpinnings of PRDM16-associated cardiomyopathies, may lead to therapeutic advances, improved patient health care and better clinical outcomes.

### 5.2 The role of PRDM16 in vascular disease: clinical studies

Several polymorphisms within the *PRDM16* locus have been associated with increased susceptibility to cerebrovascular diseases, including stroke and migraine; however, so far none of them have been labelled as pathogenic (*Table 2*).^89,90^ Migraine is a common neurovascular disease characterised by recurrent attacks of unilateral headache, often accompanied by nausea, and hypersensitivity to movement and auditory or visual inputs.^106^  ^,107^ The pathophysiologic mechanisms contributing to migraine remain incompletely understood, although cerebral vascular dysfunction has been suggested to play an important role.^108,109^ Several GWASs have associated the *rs2651899* polymorphism with common migraine,^89,98,101,104^ which was later replicated in several populations, including in Chinese,^96,99,102^ Spanish^100^ and North Indian^97^ study subjects. It is speculated that the *rs2651899* polymorphism, located within the first intron of the *PRDM16* gene, affects gene splicing or its downstream regulatory elements, which may alter *PRDM16* mRNA expression.^103^ As PRDM16 is highly expressed in vascular tissues and impaired PRDM16 function has been associated with vascular disease,^28,31^ migraine-associated *PRDM16* polymorphisms might contribute to migraine disease via the induction of vascular (dys)function,^101^ possibly via an effect on NO signalling and endothelial-dependent vasorelaxation^28^ or the oxidative stress response.^14^ This putative vascular involvement is consistent with previously reported shared polygenic risks between migraine and other cerebrovascular diseases such as stroke.^110^ Daghlas *et al.* indeed demonstrated that several genetic susceptibility loci are shared between stroke and migraine, including the known migraine susceptibility locus in *PRDM16*, although an opposite relationship was found.^105^ In support of a potential role for PRDM16 in stroke pathogenesis, a cross-ancestry genome-wise association study (GWAS) performed on stroke patients has identified a new *PRDM16* polymorphism (*rs2455132*) to be a significant risk locus for stroke, predominantly for any stroke, ischaemic stroke, and small vessel stroke.^90^ This newly identified *PRDM16* polymorphism has been predicted to alter the expression of long noncoding RNA and enhancer RNAs, mainly in endothelial and other vascular cells. However, the exact role and underlying mechanisms of this genetic *PRDM16* variant on vascular function and stroke pathogenesis remain unknown and warrant further investigation.

**Table 2:** Genetic *PRDM16* variants in patients with vascular disease

Diagnosis	Variant		Varsome^a^	Year	Type of study	Patient population	Reference
Coronary artery disease	*PRDM16 c.2604-864C > G*	*rs72633335*	NA	2017	GWAS	European population (*n* = 122 733)	^36,91b^
	*PRDM16 c.1187-1152C > G*	*rs6670123*	NA				
	*PRDM16 c.2604-240G > A*	*rs2493288*	B				
	*PRDM16 c.2604-447G > A*	*rs2493290*	NA				
	*PRDM16 c.2603 + 20C > T*	*rs2493291*	B				
	*PRDM16 c.1898C > T*	*rs2493292*	B/LB				
	*PRDM16 c.1187-916C > G*	*rs2493296*	NA				
	*PRDM16 c.1187-2036C > A*	*rs2493298*	NA				
	*PRDM16 c.37 + 25C > A*	*rs7413494*	B	2022		European population (*n* = 181 522)	^92^
BP dysregulation	*PRDM16 c.2604-864C > G*	*rs72633335*	NA	2018		European population (*n* = 120 333)	^85,93–95b^
	*PRDM16 c.1187-1152C > G*	*rs6670123*	NA				
	*PRDM16 c.2604-240G > A*	*rs2493288*	B				
	*PRDM16 c.2604-447G > A*	*rs2493290*	NA				
	*PRDM16 c.2603 + 20C > T*	*rs2493291*	B				
	*PRDM16 c.1898C > T*	*rs2493292*	B/LB				
	*PRDM16 c.1187-916C > G*	*rs2493296*	NA				
	*PRDM16 c.1187-2036C > A*	*rs2493298*	NA				
Migraine	*PRDM16 c.38-18977T > A*	*rs2651899*	NA	2011		American and European population (*n* = 5122)	^89^
				2013	Case-control association study	Chinese population (*n* = 207)	^96^
					Replication study	Indian population (*n* = 340)	^97^
					GWAS	American, Australian and European population (*n* = 23 285)	^98^
				2014	Case-control association study	Chinese population (*n* = 304)	^99^
				2015		Spanish population (*n* = 512)	^100^
				2016	GWAS	European and Australian population (*n* = 59 674)	^101^
				2017	Case-control association study	Chinese population (*n* = 581)	^102^
				2020	Meta-analysis	European, Chinese and Indian population (*n* = 2853)	^103^
				2022		European, Chinese, and Indian population (*n* = 2320)	^104^
Stroke	*PRDM16 c.38-18977T > A*	*rs2651899*	NA		GWAS	European population (*n* = 5386)	^105^
	*PRDM16 c.438 + 60382T > A*	*rs2455132*	NA			European, Asian, African American and Hispanic population (*n* = 110 182)	^90^

BP, blood pressure; GWAS, genome-wide association study.

^a^Varsome assessment^73^ of pathogenicity; if more than 1 designation is mentioned, then this reflects conflicting assessments by different studies; NA: not applicable/available; (L)B: (likely) benign variant.

^b^Original publication in case of multiple references.

Despite its established association with cerebrovascular diseases, there is only limited clinical evidence so far for a role of PRDM16 in human arterial-restricted diseases. Reduced *PRDM16* levels were observed in human atherosclerotic^37,79^ and AAA lesions,^85^ and studies in rodents reported a protective role for PRDM16 against these arterial-restricted diseases, by maintaining normal SMC function and homeostasis.^37,79,85^ Recently, several GWAS's have linked mutations in the *PRDM16* gene with arterial diseases, including CAD^91,92^ and hypertension/blood pressure dysregulation.^80,85,93–95^ Moreover, Turner *et al.* used single-nuclear ATAC sequencing to establish an association between CAD risk variants and the *PRDM16* promoter in SMCs. Furthermore, a missense CAD-associated polymorphism (*rs2493292*) in *exon 9* of *PRDM16* was identified, which was suggested to have both coding and noncoding effects on *PRDM16* expression. In addition, the *PRDM16*-regulated module was found to be highly enriched for the presence of atherosclerotic lesions and CAD severity, leading to the hypothesis that altered *PRDM16* expression in SMCs might be a key pathogenic feature and driving force of CAD.^36^ These findings are consistent with a recent study indicating enhanced atherosclerosis in mice with SMC-specific loss of PRDM16 and reduced PRDM16 protein levels in SMCs of atherosclerotic vs. healthy human arteries.^79^ While PRDM16 was mostly found in SMCs and less so in ECs of healthy human coronary arteries,^79^ in early-stage atherosclerotic arteries its expression was more restricted to the *vasa vasorum* and endothelium.^36^ Interestingly, in more advanced stages of human coronary atherosclerosis, PRDM16 protein levels were markedly reduced within the endothelium,^36,37^ again demonstrating the multicellular role of PRDM16 in arterial health and disease.

## 6. Conclusion and future perspectives

Recent evidence has highlighted the indispensable role of PRDM16 within the cardiovascular system where it—in concordance with its unique expression profile—coordinates ventricular and arterial shape and function. These studies have also indicated the multi-modal mechanism via which PRDM16 coordinates cardiovascular development, via either (in)direct binding to the DNA as a TF, or by acting as an epigenetic (co-)factor, thereby modulating canonical pathways such as NOTCH and TGF-β signalling. In addition to PRDM16's role during development, the observations made by us and others unequivocally point towards its protective role against several CVDs, including cardiomyopathies, atherosclerosis, AAA, and PAD. Moreover, the gene dose-dependent effect of PRDM16 loss in mice suggests that therapeutically increasing PRDM16 levels could significantly promote cardiovascular health and improve disease outcomes.^60^

In case we envisage boosting PRDM16 expression/activity directly, we need to identify upstream regulators of its expression and/or rely on posttranslational modifications (PTMs) that stabilise the PRDM16 protein. Upstream regulators of *PRDM16* expression remain however largely unknown. We and others demonstrated that arterial ECs in culture no longer express PRDM16, suggesting that, at least within the endothelium, microenvironmental factors—the nature of which needs to be determined—may significantly drive its expression.^27^ Several microRNAs are known to modify PRDM16 expression in adipose tissue (reviewed in Ref.^111^) and testing their effect in arterial ECs and SMCs might provide an alternative avenue to boost PRDM16 expression. However, future studies using PRDM16 reporter cells, mice, or zebrafish are urgently needed to dissect the upstream regulators of PRDM16 in the heart and vasculature or identify small molecule compounds that boost its expression. Unlike its upstream regulators, the PTMs that prevent PRDM16 protein degradation and increase its stability are well-known, but whether these PTMs can be leveraged to promote cardiovascular health remains to be proven, as most have been documented in adipose tissue (reviewed in Ref.^111^). One of the drugs currently known to promote the stability of PRDM16 is the anti-diabetic drug rosiglitazone (reviewed in Ref.^9^); however, its long-term use has been associated with increased risk for myocardial infarction. Hence, carefully analysing and correlating the cardiovascular levels of PRDM16 with disease severity and corresponding drug regimen is required to fully understand the potential therapeutic effect of PRDM16 modulators. As an alternative, a more tailored approach could be envisioned in which, with the help of rapidly evolving omics technologies, the molecular mechanisms—beyond the known NOTCH and TGF-β signalling pathways—activated downstream of PRDM16 during each of the abovementioned CVDs are unravelled and targeted.

Finally, the ability of PRDM16 to determine cell fate in CMs, SMCs, and ECs may offer the potential for optimising differentiation protocols based on iPSCs in order to obtain more pure populations of ventricular working CMs, arterial SMCs, and arterial ECs, respectively. These refined cell types may be used for drug screening purposes or implemented in advanced regenerative approaches.

## Data Availability

No data were generated in this manuscript.
